# Effects of Thermal-Strain-Induced Atomic Intermixing on the Interfacial and Photoluminescence Properties of InGaAs/AlGaAs Multiple Quantum Wells

**DOI:** 10.3390/ma16176068

**Published:** 2023-09-04

**Authors:** Zhi Yang, Shuai Zhang, Shufang Ma, Yu Shi, Qingming Liu, Xiaodong Hao, Lin Shang, Bin Han, Bocang Qiu, Bingshe Xu

**Affiliations:** 1 Materials Institute of Atomic and Molecular Science, Shaanxi University of Science and Technology, Xi’an 710021, Chinazhangshuai20210830@163.com (S.Z.);; 2 School of Materials Science and Engineering, Shaanxi University of Science and Technology, Xi’an 710021, China; 3 School of Fashion and Textiles, The Hong Kong Polytechnic University, Hung Hom, Kowloon, Hong Kong SAR, China; 4 Key Laboratory of Interface Science and Engineering in Advanced Materials of Ministry of Education, Taiyuan University of Technology, Taiyuan 030024, China

**Keywords:** InGaAs/AlGaAs, thermal strain, quantum-well intermixing, interfacial quality, wavelength blueshift

## Abstract

Quantum-well intermixing (QWI) technology is commonly considered as an effective methodology to tune the post-growth bandgap energy of semiconductor composites for electronic applications in diode lasers and photonic integrated devices. However, the specific influencing mechanism of the interfacial strain introduced by the dielectric-layer-modulated multiple quantum well (MQW) structures on the photoluminescence (PL) property and interfacial quality still remains unclear. Therefore, in the present study, different thicknesses of SiO_2_-layer samples were coated and then annealed under high temperature to introduce interfacial strain and enhance atomic interdiffusion at the barrier–well interfaces. Based on the optical and microstructural experimental test results, it was found that the SiO_2_ capping thickness played a positive role in driving the blueshift of the PL peak, leading to a widely tunable PL emission for post-growth MQWs. After annealing, the blueshift in the InGaAs/AlGaAs MQW structures was found to increase with increased thickness of the SiO_2_ layer, and the largest blueshift of 30 eV was obtained in the sample covered with a 600 nm thick SiO_2_ layer that was annealed at 850 °C for 180 s. Additionally, significant well-width fluctuations were observed at the MQW interface after intermixing, due to the interfacial strain introduced by the thermal mismatch between SiO_2_ and GaAs, which enhanced the inhomogeneous diffusion rate of interfacial atoms. Thus, it can be demonstrated that the introduction of appropriate interfacial strain in the QWI process is of great significance for the regulation of MQW band structure as well as the control of interfacial quality.

## 1. Introduction

A strained InGaAs/AlGaAs multiple quantum well (MQW) system as the active region of semiconductor materials has been widely applied in lasers, detectors, and various optoelectronic devices based on its excellent properties of thermal stability and wide range of tunable wavelength [1,2,3,4]. Furthermore, an effective methodology for the modification of the bandgap energy also plays a significant role in the fabrication of these relevant monolithic integration optoelectronic components. Therefore, existing studies have developed several technical approaches to adjust the bandgap energy of quantum wells (QWs), such as ion-irradiation-induced disordering [5], plasma-damage-induced disordering (PID) [6], impurity-induced disordering (IID) [7], and impurity-free vacancy disordering (IFVD) [8]. Compared with other techniques, IFVD is generally considered as a simple and flexible production process for the tuning of the emission wavelength in QW systems [9]. Commonly, after the thin dielectric layer is coated around the QW surface and the rapid thermal annealing (RTA) process is carried out, selective regional adjustment of the bandgap energy can be achieved based on the IFVD technique [10]. However, the intermixing atomic treatment in IFVD leads to major changes of scale in the shape, dimension, and composition of the QWs, which have crucial impacts on the optical absorption and emission characteristics of the integration devices [11,12,13,14]. Therefore, the appropriate selection of the atomic intermixing conditions needs to be explored scientifically to obtain better photoelectric performances in IFVD-induced QWs.

Although the majority of researchers have demonstrated that strain-enhanced interdiffusion is an efficient strategy to expand the QW emission wavelength range, recently, some of them also consider that strain-introduced crystal defects could improve the intermixing diffusion rate to some extent [15,16]. However, the exact working mechanism is still controversial. Particularly during the high-temperature annealing process, atoms diffuse between the barrier and the well interface and then form a nonabrupt interfacial structure, resulting in exciton transition to a higher level. Generally, the factors that affect the emission wavelength of the intermixed region include not only the interfacial disorder but also the interfacial strain. For instance, the thermal expansion coefficient mismatch between the film and the semiconductor introduces strong interfacial strain and thus promotes effective bandgap drift [17,18]. Previous studies have adopted photoluminescence (PL) to analyze the intermixing degree of interfacial atoms by considering the change in the diffusion components rather than the exact effect of the interfacial strain and the microstructure on their optical properties [9,19]. Recently, we obtained the interfacial microstructure of epitaxial wafers by transmission electron microscopy and explained the effects of interfacial atomic segregation as well as interstitial atoms on the luminescence properties of QWs [20,21,22,23]. Therefore, the influence of strain-induced intermixing on the QW luminescence characteristic is complex and needs to be further explored. 

Consequently, we aimed to explore the strain-induced effects on the luminescence properties and microstructural interfacial characteristics in intermixed InGaAs/InGaAs MQWs. QW samples were coated with various thicknesses of SiO_2_ layers and diffused using the rapid annealing treatment. The optical performances as well as the structural and strain-related characteristics were tested using room-temperature photoluminescence (PL), high-resolution X-ray diffractometry (HRXRD), and spherical aberration-corrected scanning transmission electron microscopy (ACTEM), respectively. The experimental results indicate that the PL peak shift can be achieved at 30 eV. The apparent strain-induced blueshift behavior and the bending phenomenon of the well-layer fringe in the QWs were observed clearly after the intermixing treatment, which was related to the propagation of elastic strain and the nonuniform atomic diffusion behavior. Therefore, the present study not only promotes the scientific understanding of the interaction between the interfacial strain and the optical properties in QW, but it also provides fundamental references for the studies of induced-strain intermixed conditions and even the optimization of QW structural characteristics.

## 2. Experimental Section

**Figure 1:**
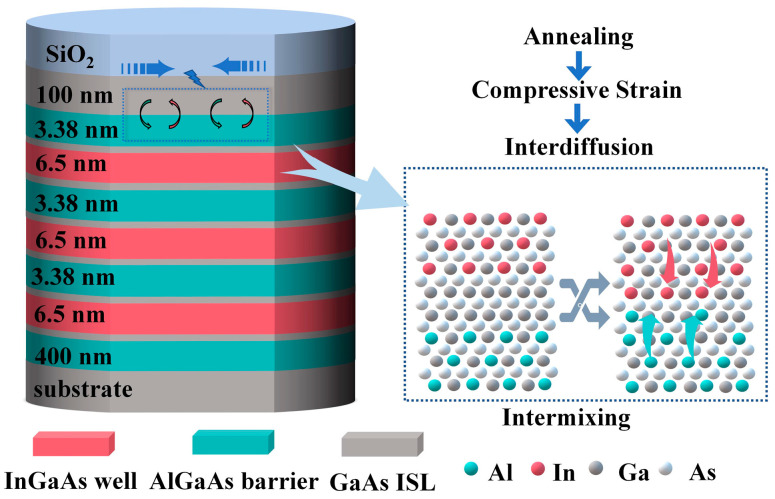
InGaAs/AlGaAs MQW sample with SiO_2_ capping layer and the atomic interdiffusion process at the barrier–well interfaces under high-temperature annealing.

Epitaxial growth: InGaAs/AlGaAs MQWs were grown through metal–organic chemical vapor deposition (MOCVD, AIXTRON 3 × 2) on an n-type GaAs substrate, and the cross-sectional views of the experimental samples are as shown in Figure 1. In this study, trimethylgallium, trimethylindium, trimethylaluminum, and arsine were used as the precursor. The epitaxial structure consisted of a 400 nm thick Al_0.3_Ga_0.7_As buffer layer, three pairs of MQWs with a 0.56 nm GaAs layer inserted between the 6.50 nm In_0.15_Ga_0.85_As well layer and the 3.38 nm Al_0.3_Ga_0.7_As barrier layer and a 100 nm GaAs capping layer. 

Capping and annealing: Before the annealing treatment, SiO_2_ dielectric capping layers with a thickness of 200 nm and 600 nm were deposited, respectively, on the as-grown sample surface through the electron beam evaporation technique. Then, the 0 nm, 200 nm, and 600 nm SiO_2_-coated as-grown samples were rapidly annealed in a nitrogen ambience at 850 °C for 180 s and labeled RTA@0, RTA@200, and RTA@600, respectively. For comparison, the as-grown sample without heat treatment was labeled Non-RTA@0. And, to avoid arsenic atom escape from the surface, the annealed samples were covered with a layer of SiO_2_. 

Material characterization: The optical properties were investigated by applying PL (HORIBA iHR320) at room temperature with a laser emission wavelength of 532 nm. Then, a high-resolution X-ray diffraction (HRXRD, Bruker D8) instrument was adopted to measure the crystal quality and strain variations due to the SiO_2_ coating and annealing processes. Moreover, aberration-corrected transmission electron microscopy (ACTEM JEOL ARM-300F) was applied to observe the diversity of the interfacial structure at a 300 kV acceleration voltage.

## 3. Results and Discussion

**Figure 2:**
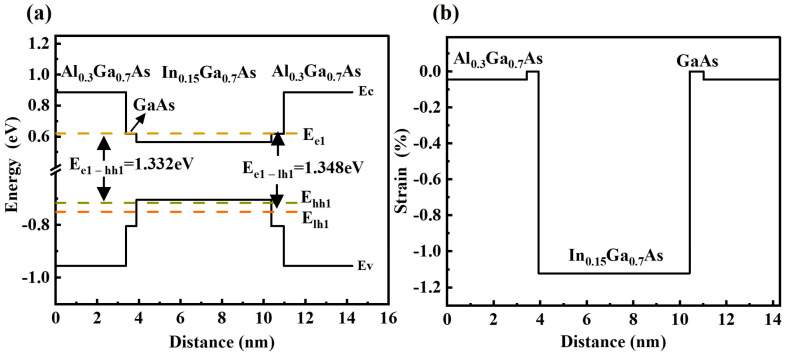
Nextnano++ (**a**) energy band structure, electron–hole energy levels and (**b**) elastic strain simulations of as-grown sample at 300 k. The Ec, Ev, E_e1_, E_hh1_, and E_lh1_ are the energies of the conduction band, the valence band, the electronic ground state, and the heavy and light hole bands, respectively.

Prior to discussing the experimental results, taking into account the theoretical energy band structure of the fabricated samples facilitates the understanding of the photogenerated carriers’ exact optical transitions. The Nextnano++ 3.1.0 software was employed to simulate the properties of the MQW materials under 300 k conditions, including band-edge band structure, electron–hole localization, and strain, based on the structural parameters of the InGaAs/AlGaAs MQWs grown as in Figure 1. The material parameters of GaAs, InAs, and AlAs, including Eg and the electron effective mass, were taken from the database that comes with the software, and Vegard’s theorem was employed to calculate the energy-band edge structure of the well-layered and barrier-layered ternary alloys. The energy band shifts between the well and barrier layers were obtained through simulations based on a self-consistent solution of the Schrödinger equation taking into account the effect of band-edge shifts induced by the InGaAs/AlGaAs strain, as shown in Figure 2. In addition, the strain also plays an important role in separating the subband energies of the quantum wells. For InGaAs/AlGaAs MQWs, the compressive strain increases the compressive strain by more than 1.0%, and the valence interband and splitting are the key factors affecting the position of the HH and LH band edges.

**Figure 3:**
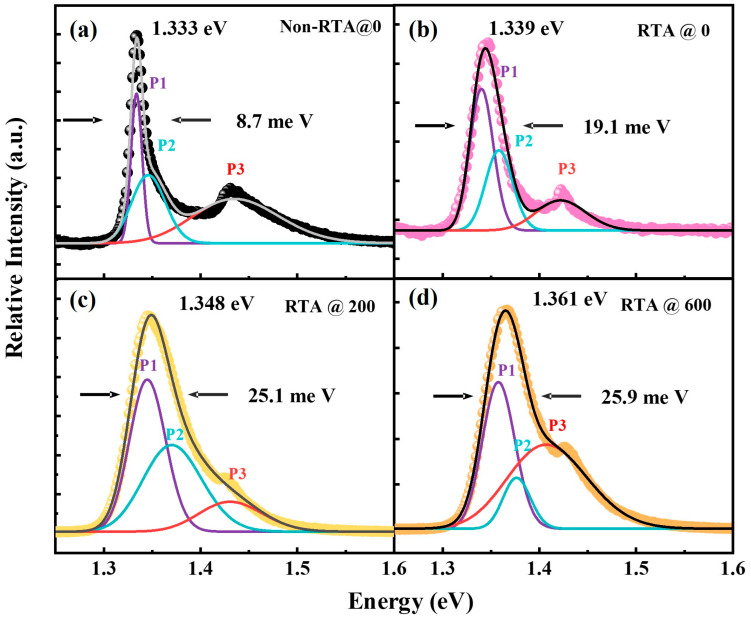
The room-temperature PL spectra and Gaussian fitting curves for (**a**) Non-RTA@0, (**b**) RTA@0, (**c**) RTA@200, (**d**) RTA@600.

The normalized PL intensity spectra of the fabricated samples are shown in Figure 3 for comparison. Based on the Gaussian fitting curves, the emission spectra of all samples consisted of three components, where the transition energies of the main and shoulder peaks are related to the valence band splitting of the heavy hole (*HH*) band and light hole (*LH*) band generated by quantum confinement. The main peak (P1) is associated with the radiative recombination of ground-state electrons and heavy holes, and the shoulder peak (P2) is related to the radiative recombination of ground-state electrons and light holes [24]. The peak (P3) located at ~1.425 eV is considered as the PL peak of the GaAs substrate material. From Figure 3, we can see the blueshift behavior of the InGaAs/AlGaAs MQWs after annealing as their emission peak with the SiO_2_ capping layer shifted from 1.333 eV to 1.361 eV compared with the non-capped samples. Additionally, on increasing the SiO_2_ thickness from 0 nm to 600 nm, the full width at the half-maximum (FWHM) of the PL spectrum was found to be broadened from 8.7 meV to 25.9 meV. It can also be observed that the unnormalized PL intensity signal of all the annealed coating samples became weaker. Firstly, group III vacancies were created at the GaAs–SiO_2_ interface due to the out-diffusion of Ga atoms into the dielectric capping layer [16]. The group III vacancy diffused into the QW region, promoted the atomic interdiffusion between the barrier and the well, and therefore, led to the PL peak shift toward the higher-energy side. Secondly, due to the difference in thermal expansion coefficients between SiO_2_ (5.2 × 10^−7^ °C^−1^) and GaAs (6.86 × 10^−6^ °C^−1^) by an order of magnitude, compressive stress was generated at the GaAs/SiO_2_ interface to affect the emission wavelength during the annealing process [25,26]. Thirdly, as the compressive strain increased, the conduction-band edge grew and the valence-band edges shrank conversely; consequently, the bandgap became wider, and the emission peaks tended to display a large blueshift. Thus, the blueshift of the bandgap is related to the changes in material composition as well as the induced strain during the intermixing process. The broadening and weakening of the emission peak can be attributed to the nonradiative recombination effect caused by the QW structural disorder. It can be demonstrated that the blueshift behavior of the InGaAs/AlGaAs MQWs can be effectively enhanced by increasing the thickness of the SiO_2_ capping layer.

**Figure 4:**
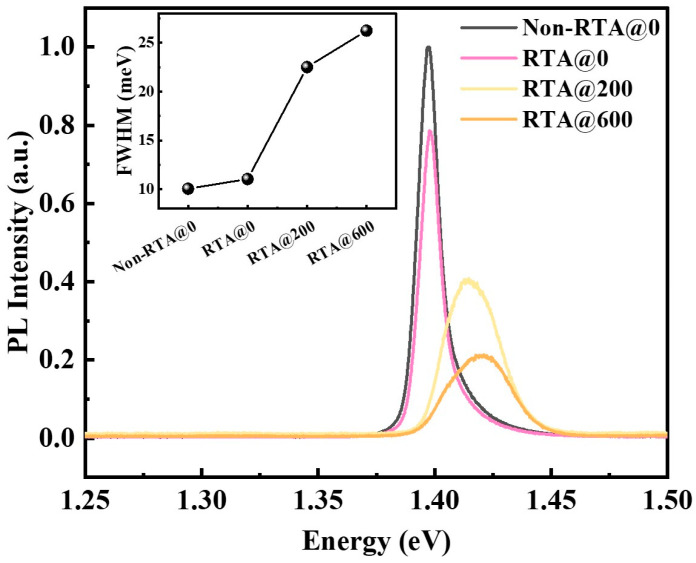
The PL curves for all samples measured under 77 k conditions. The inset graph illustrates the variations in the FWHM of the PL curves for each of the samples.

To verify the effect of interfacial atomic diffusion on the microstructure and photoluminescence properties of InGaAs/AlGaAs MQWs, all samples were measured using PL at 77 K; the results are shown in Figure 4. The peak intensity of sample RTA@0 was slightly lower as compared to that of sample Non-RTA@0, and the PL peak position was nearly unchanged. In general, the diffusion of atoms at the quantum-well interface was not activated by a short rapid annealing. In contrast, the intensity of the RTA@600 sample decreased rapidly and the half-width more than doubled under the same thermal treatment conditions. Atom diffusion at the quantum-well interface disrupts the atomic structure of the quantum well and increases the interface alloy disorder, which increases the scattering chance of carriers. The results of the 77 K PL further confirm that the introduction of the SiO_2_ layer can effectively enhance the mixing of interfacial atoms to tune the quantum emission wavelength.

**Figure 5:**
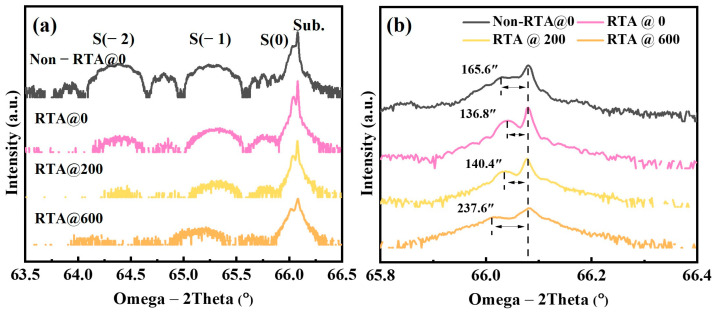
(**a**) HRXRD ω/2θ (004) scans of the MQWs, (**b**) substrate and zeroth-order satellite peaks of enlarged areas.

The variations in the structural characteristics and strain of the annealed MQWs with different thicknesses of SiO_2_ capping layers were investigated through the HRXRD spectrum, as illustrated in Figure 5. The observed clear interference fringes in the Non-RTA@0 sample suggest that these MQWs maintained excellent interfacial crystal quality and periodicity. For all annealed samples, the intensity of the satellite peak decreased and was accompanied by the disappearance of the secondary satellite peak, which indicated the slight degradation of the abrupt interface of the InGaAs/AlGaAs MQWs. Moreover, the level-zero satellite peaks of the annealed samples moved closer to and then further away from the GaAs substrate peaks based on the increasing thickness of the dielectric coatings, which means that the strain of the MQWs changed. The values of the zeroth-order satellite peak away from the GaAs peak for the four samples were 165.6, 136.8, 140.4, and 237.6 arc-seconds, respectively. The quantitative interfacial strain value in the InGaAs/AlGaAs MQWs can be calculated using the following formula [27]:

(1) $$\varepsilon =cot\theta_{e}∆\theta$$

where ε is the actual strain of the InGaAs/AlGaAs MQWs, $\theta_{e}$ is the Bragg angle of the GaAs substrate, and ∆θ is the separation between the zeroth-order satellite peak and the GaAs substrate [28]. Then, the actual strain in the four samples was −1.23 × 10^−3^, −1.05 × 10^−3^, −1.02 × 10^−3^, and −1.77 × 10^−3^, respectively; here, the negative sign accounts for compressive strain. For the RTA@0 sample, the strain was significantly degraded compared with that of the Non-RTA@0 sample, which can be interpreted as the relief of the annealed strain. In contrast, the increased strain in the RTA@600 sample can be considered the result of the competition between the strain introduced by both the SiO_2_ capping and the relief stress during the annealing process [29]. As mentioned above, it was found that the atomic intermixing at the strain-enhanced interface was the main reason for the strain variation and the disappearance of the satellite peaks.

**Figure 6:**
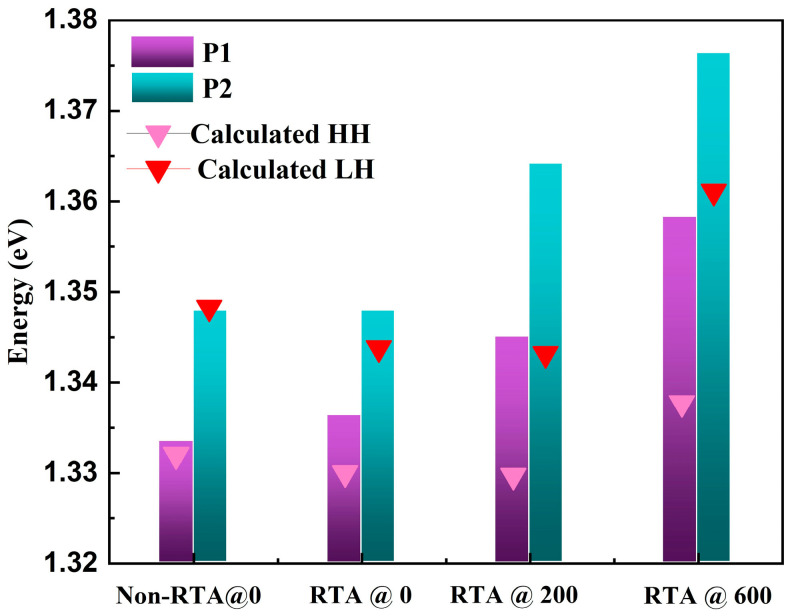
The evolution of P1, P2 and the calculated values of HH and LH emission for different samples. The triangle symbols are calculated results for InGaAs/AlGaAs MQWs.

In order to theoretically investigate the energy bandgap blueshift mechanism under induced interfacial strain, quantitative calculations were carried out, taking quantum confinement effect into account, to determine the relationship between the emission peak blueshift and the strain. The conduction band of heavy holes (*HHs*) and light holes (*LHs*) in the InGaAs well layer under the strain condition were obtained using the following equations [30]:

(2) $$E_{c-HH}=E_{0}\left({In}_{x}{Ga}_{1-x}As\right)+2a\left(1-\frac{C_{12}}{C_{11}}\right)\varepsilon -b\left(1+2\frac{C_{12}}{C_{11}}\right)\varepsilon$$

(3) $$E_{c-LH}=E_{0}\left({In}_{x}{Ga}_{1-x}As\right)+2a\left(1-\frac{C_{12}}{C_{11}}\right)\varepsilon +b\left(1+2\frac{C_{12}}{C_{11}}\right)\varepsilon$$

(4) $$a=a_{c}-a_{v}$$

where *E*_0_*(In_x_Ga*_1−*x*_*As*) is the unstrained bandgap of the bulk InGaAs material, assuming that the InGaAs bandgap at 300 k follows the relation *E*_0_(*x*) = (1.422 − 1.53*x* + 0.45*x*^2^)eV, where *x* is the indium mole fraction [31]. Moreover, *a_c_* and *a_v_* are the conduction- and valence-band deformation states, *b* is the shear deformation state, *C*_11_ and *C*_12_ are the elastic-stiffness constants; their offsets relative to the indium content were calculated through linear interpolation as described in Refs. [32,33,34]. Considering the effect of quantum confinement on the transition energy level of the carrier, the values of *HHs* and *LHs* in the well were calculated using the Schrodinger equation according to the boundary conditions of the wave function. Based on the above equations, the obtained transition energy levels of *HHs* and *LHs* at 300 k are plotted in Figure 6.

The theoretical calculated values of all the annealed samples were smaller than the corresponding experimental results (Figure 6). In addition, the wavelengths obtained based on the elastic strain theory exhibited a trend of redshift and then blueshift as the thickness of the SiO_2_ capping layer increased. For RTA@200, although the actual strain was almost the same as that of RTA@0, its emission peak had a relatively larger blueshift than the experimental tested results. In general, the intermixing rate in the MQWs mainly depends on the diffusivity and concentration of group III vacancies. The strain in RTA@200 was insufficient to generate a significant band shift; therefore, the principal factor for the blueshift was the increasing number of cation vacancies caused by the SiO_2_ capping. Notably, an obvious blueshift phenomenon in the RTA@600 sample with a thicker capping layer was observed due to the interfacial-strain-induced enhancement of the bandgap in the InGaAs/AlGaAs MQWs.

**Figure 7:**
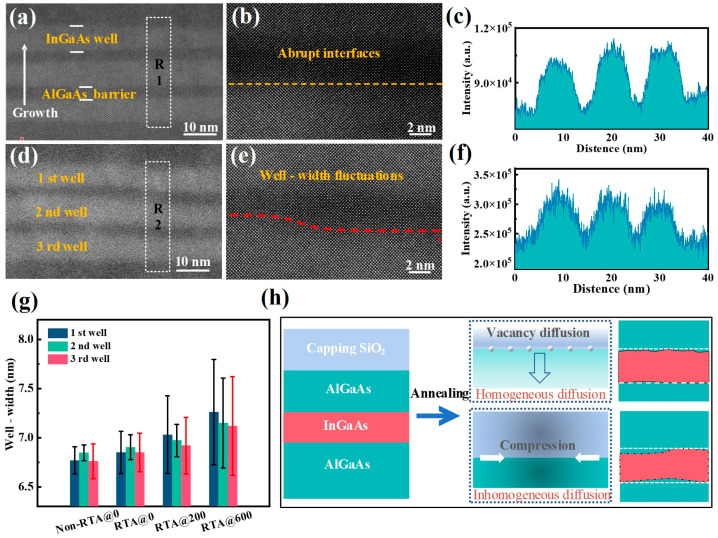
Cross-sectional HAADF images of the samples (**a**,**b**) Non-RTA@0 and (**d**,**e**) RTA@600. The higher magnifications of the white boxes in figure (**a**,**d**) are displayed in (**c**,**f**). (**g**) The average thicknesses of different well layers in the samples were measured at 10 different areas; the error bar represents the standard deviation of the well width. (**h**) Well-layer shape deformation in InGaAs/AlGaAs MQWs.

To figure out the interfacial microstructure and quality of the InGaAs/AlGaAs MQWs with a higher resolution, high-angle annular dark field (HAADF) images were plotted. Figure 7a,d show the cross-sectional HAADF images of the Non-RTA@0 and RTA@600 samples, respectively. As can be observed, the well layer and barrier layer in the as-grown sample were neatly arranged as a high-quality abrupt interface. Compared to Figure 7b, the wells fluctuated significantly, and their interfaces became gradually blurred in Figure 7e (RTA@600 nm). Figure 7c,f show the atomic column intensity profiles curves along the growth direction in the Non-RTA@0 and RTA@600 samples, respectively. These profiles show that the intensity contrast oscillations between the wells and barriers were gradually flattening, indicating that the compositional variations between the wells and barriers turned from an abrupt distribution to a gradient distribution. In addition, the well-width fluctuation penetrated from the surface to the inside of the active region and propagated vertically through the entire quantum-well structure. We expect that the width fluctuation may be caused by the nonuniform diffusion of atoms between the well–barrier interfaces. As illustrated in Figure 7g, the modulation amplitudes of the well thickness tended to decrease from the surface to the penetration direction of the substrate. Generally, the expansion of the well width causes the redshift of the PL spectrum to a certain degree; however, the diversities of the barrier and well components due to atomic diffusion and the bandgap modifications caused by the interfacial strain are also important factors affecting the PL luminescence properties. Finally, the thickness fluctuations reflect the deterioration of the optical and interfacial quality, and this is responsible for the degradation of the HRXRD satellite peaks and PL intensity.

The influences of the high-temperature annealing process on the InGaAs/AlGaAs MQWs can be categorized into interdiffusion and strain effects, with the schematics of well-width fluctuation illustrated in Figure 7h. Regarding the interdiffusion effects, during the high-temperature annealing process, the vacancy on the GaAs surface diffuses into the QW region, leading to a decrease in the amplitude distribution of the barrier-width fluctuation, while the upper QW interface appears rougher than the lower interface. Considering the strain effects, the thermal expansion coefficient mismatch between the SiO_2_ capping layer and the GaAs layer induces a high strain field gradient, whereas the strain field perpendicular to the growth direction exerts stress on the quantum well and distorts the striations in the QW region. Based on the above reasons, the induced strain and the inhomogeneous diffusion of vacancies are responsible for the blurring of the interface and the gradient distribution of well-width fluctuations.

## 4. Conclusions

In summary, we systematically explored the influences of the thickness of an SiO_2_ capping layer on the PL properties and interfacial structural characteristics of InGaAs/AlGaAs MQWs. According to the tested results, the wavelength blueshift amplitude of the annealed MQWs improved on the increase of the capping thickness, which indicates that the interfacial strain introduced by the thermal mismatch between the dielectric layer and the semiconductor can not only enhance the interfacial atomic intermixing but also further improve the tunability of the PL peak for MQWs. Moreover, the inhomogeneous atomic diffusion during the propagation of interfacial strain led to the degradation of the interfacial quality, which was also confirmed by the variation in well-width fluctuation observed in the HAADF images and XRD patterns after annealing treatment. Therefore, the studied findings demonstrated the relationships among introduced strain, PL properties, and interfacial structure during interface mixing, which provides a new idea for the development of wavelength-tunable photonic integration devices with excellent performances in the semiconductor field.

## Data Availability

The data used to support the findings of this study are available from the corresponding author upon request.
